# Supplementary material for: Self-clocking fast and variation tolerant true random number generator based on a stochastic mott memristor
Source: Nat Commun. 2021 May 18;12:2906. doi: 10.1038/s41467-021-23184-y (PMC8131590; doi:10.1038/s41467-021-23184-y)
Supplement: Supplementary file 1 — Supplementary Information [file 41467_2021_23184_MOESM1_ESM.pdf]

Supplementary Information

Self-clocking fast and variation tolerant true random number generator based on a stochastic mott memristor

Gwangmin Kim[1], Jae Hyun In[1], Young Seok Kim[1], Hakseung Rhee[1], Woojoon Park[1], Hanchan Song[1], Juseong Park[1] and Kyung Min Kim[*]

[1]Department of Materials Science and Engineering, Korea Advanced Institute of Science and Technology (KAIST), 291 Daehak-ro, Yuseong-gu, Daejeon 34141, Republic of Korea

G. Kim and J. H. In contributed equally to this work.
*Correspondence and requests for materials should be addressed to K.M.K. (email: km.kim@kaist.ac.kr).

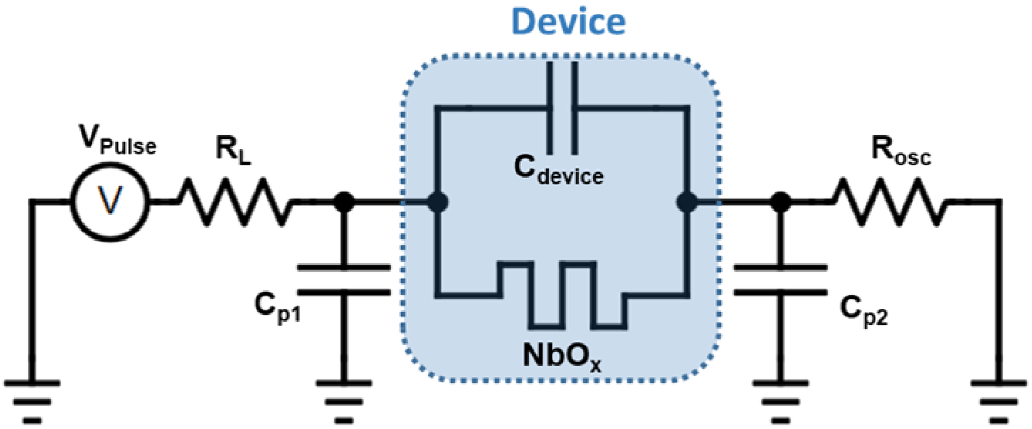

Figure S1 | Detailed oscillator circuit scheme. In this configuration, the C_{p1} and C_{p2} are the parasitic capacitance by electrical measurement setup or device geometry. The C_{device} is the NbO_x TS device capacitance, R_{osc} is a 50 ohms-resistor in the oscilloscope, and the R_L is the load resistor connected with the device in series. For numerical simulation, we used the capacitance values, 42pF, 42pF, and 85pF for C_{p1}, C_{p2}, and C_{device} respectively.

The parasitic capacitance in the breadboard is ~ 1 pF which is very low compared to the capacitors above. The specification of op-amp says, it will take 40 ns for it to successfully amplify the input (We used NE55322P op-amp having 9V/µs slew rate. Typical ΔV_{NDR} (V_{th} - V_h) is ~ 0.15 V, so the amplifier should change its output $2\Delta V_{NDR}$ multiplied by 1.2 which is the amplification factor determined by $1+R_f R_s^{-1}$ (R_f = 200Ω, R_s=1kΩ in Fig. 4)). Also, the it takes about 16 ns for a typical flip-flop to convert its output low-to-high level, and 25 ns for vice versa. The oscillation frequency ranges ~ MHz, so the peripheral components do not limit the TRNG operation as they operate at high speed.

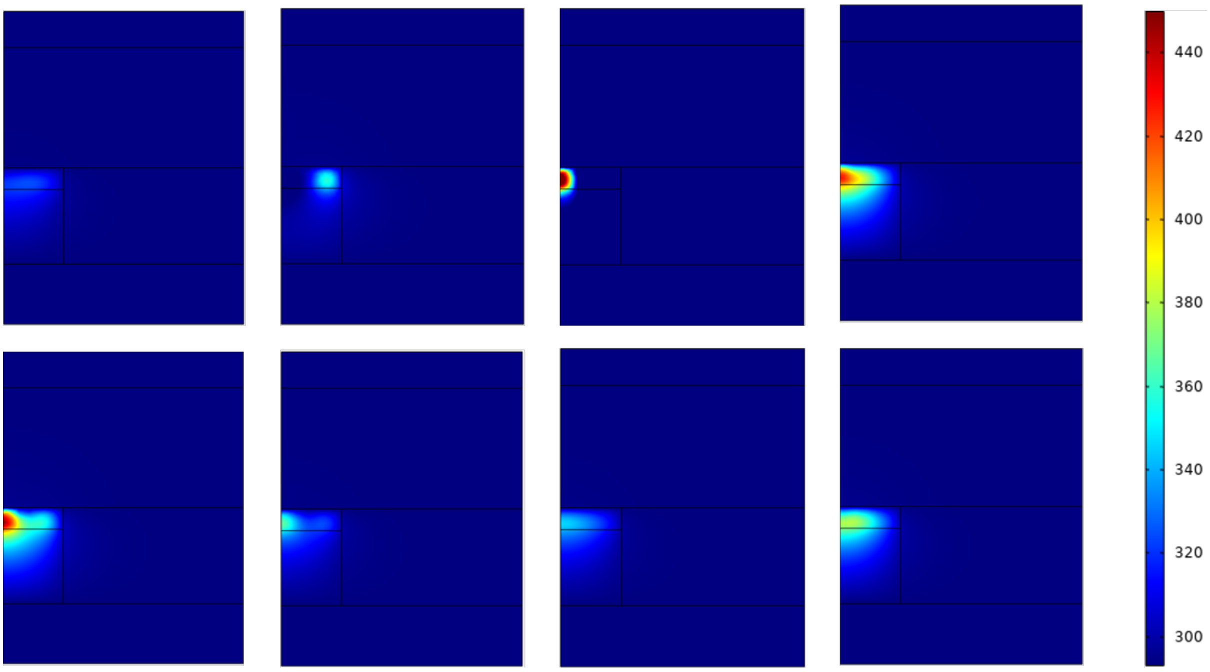

Figure S2 | Variation of heat distribution at the moment of threshold switching. The 8 heat maps were captured at the moment of the threshold switching, point 2 in Fig. 2e, during 8 pulse cycles. The simulation showed that not only the maximum temperature but also its position was changed at the moment of threshold switching. Such non-uniform variation of the heat can explain the thermal fluctuation during the switching cycle.

Specification comparison table of volatile-memristor-based TRNGs

	Bit generation rate (µs/bit)	TS memristor			Clock generator		Other active components	
		Power		Energy consumption (nJ/bit)	Clock generator power	Energy consumption (nJ/bit)	Total number (List)	Energy consumption (nJ/bit)
		Operating voltage (V)	Operating current (mA)					
Jiang, H. *et al.* [1]	**166.6**	0.4	10^{-5}	**0.8×10^{-3}**	~150 mW	**~2.5×10^{4}**	4 (Comparator, AND gate, Counter × 2)	Each active component consumes ~ mW.
Woo, K. S. *et al.* [2]	**62.5**	10	10^{-6}	**3.15×10^{-3}**	~150 mW	**~9.4×10^{3}**	6 (XNOR, XOR gates, D flip-flop × 4)	
This work	**25**	1.45	0.144 (in average during oscillation)	**5.22 (including the clock)**	-	**0**	2 (Op-amp, T flip-flop)	

Figure S3 | The energy consumption comparison table between volatile-memristor based TRNGs. The clock generator is the highest energy-consuming part. A typical ultra-low-power clock generator, CDCI6214 by Texas Instruments, consumes 150 mW of power. The clock generator should be active during the entire bit generation time. Thus, they consume about 25 μJ/bit in ref. 1 and 9.4 μJ/bit in ref. 2, which is far higher than the energy consumption in the TS memristor. Our TRNG does not require the external clock generator due to its inherent self-clocking characteristic, allowing a significant energy consumption reduction. Moreover, the number of other active components is also the minimum, confirming our TRNG is the most compact and energy-efficient.

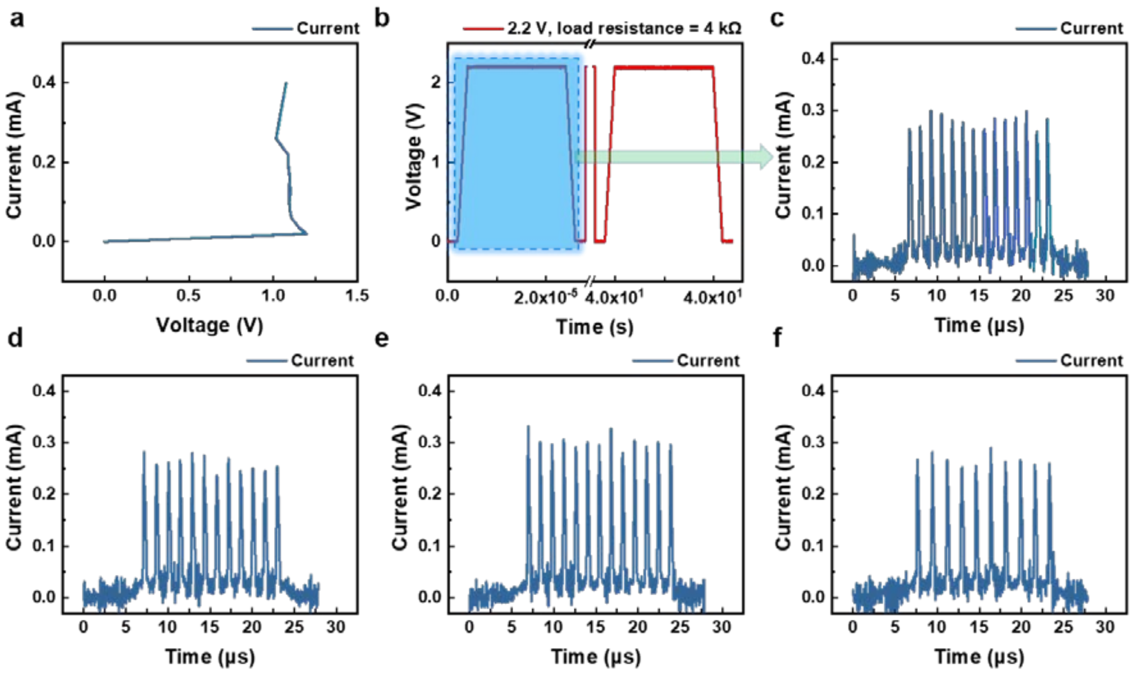

Figure S4 | Endurance characteristics of the NbOx-based mott device. For endurance testing, we applied a constant DC voltage to the NbOx-based oscillator and monitored the oscillating pulses per every 40 seconds with a load resistance of 4 kΩ. **a** shows the I – V curve of the device used for endurance test. **b** shows three pulse trains (reading – cycling – reading) whose widths are 20 μs, 40 s, and 20 μs, respectively. The pulse amplitude was 2.2 V. The reading pulse verifies whether the oscillator works or not. The cycling pulse operates the device for several million times approximately. The oscillation data at the reading period after **c** 120 s, **d** 240 s, **e** 480 s, and **f** 600 s are shown. The number of switching was calculated by the following equation; $f_{avg,s} \times t$, where $f_{avg,s}$ is the average oscillation frequency of 0.67 MHz, and t is 600 seconds. It gives 4×10^8 cycles. Our TRNG can generate one bit per 25 μs. Thus, it can generate at least 2.4×10^7 random bits per device, which is reasonably high for the practical application.

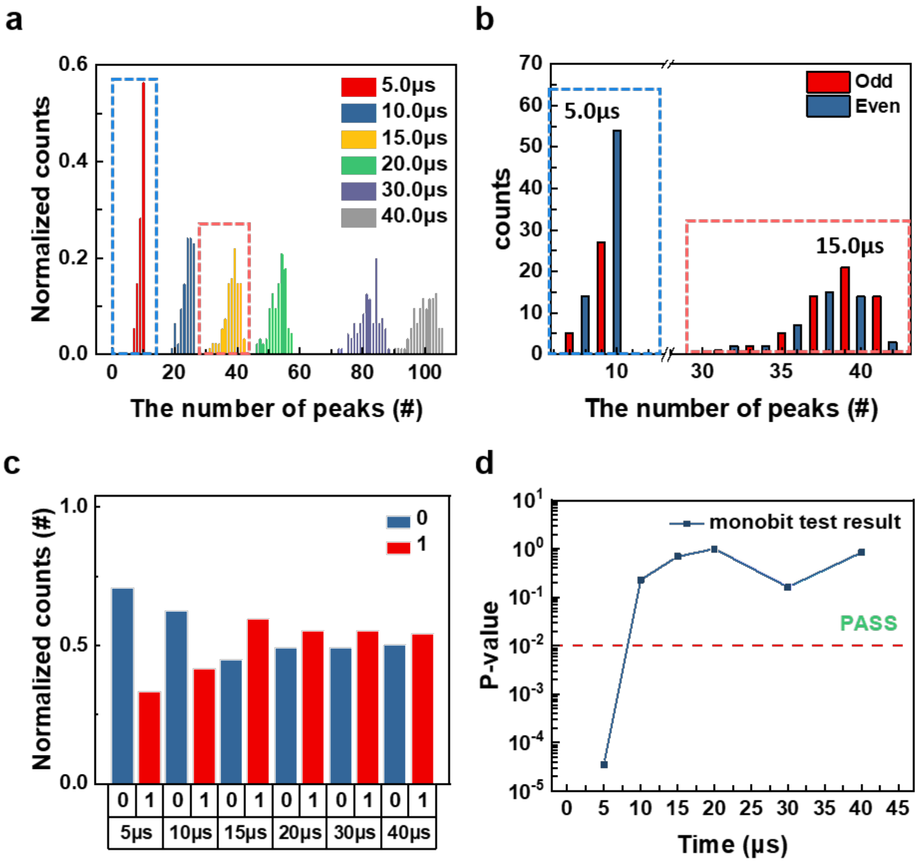

Figure S5 | Time-variant characteristic of the accumulation of stochasticity. a The distribution of the total number of peaks at given pulse times. Each distribution was obtained from 100 cycles. **b** The distribution of the number of peaks of 5 μs and 15 μs cases in **a**. It distinguishes the expected output, whether 1 or 0, as a function of the number of peaks. At a 5 μs of input pulse time, the number of peaks was ranging from 7 to 10, which is narrow. Also, their distribution was not uniform. Therefore, the 0 output was more frequent than 1, which was mainly determined by the majority case, 10. Whereas, at a 15 μs of input pulse time, the number of peaks was distributed broadly, ranging from 31 to 42. In this case, the portion of the odd and even numbers can be more random. **c** A histogram showing the portion of 0 and 1 outputs for various input pulse time. At 5 μs and 10 μs pulse, the portion of '0' is higher than '1', inferring they are not true random numbers. The portion gets closer to 0.5 as the input pulse time gets longer, leading to the true random number generation. **d** The P-value of the mono-bit test using the data of **c**.

References

1 Jiang, H. *et al.* A novel true random number generator based on a stochastic diffusive memristor. *Nat Commun* **8**, 882 (2017).

2 Woo, K. S. *et al.* A Combination of a Volatile - Memristor - Based True Random - Number Generator and a Nonlinear - Feedback Shift Register for High - Speed Encryption. *Advanced Electronic Materials* **6**, 1901117 (2020).
