# Supplementary material for: Self-clocking fast and variation tolerant true random number generator based on a stochastic mott memristor
Source: Nat Commun. 2021 May 18;12:2906. doi: 10.1038/s41467-021-23184-y (PMC8131590; doi:10.1038/s41467-021-23184-y)
Supplement: Supplementary file 3 — Description of Additional Supplementary Files [file 41467_2021_23184_MOESM3_ESM.pdf]

Title: Supplementary Movie 1

Description: The video recoded experimental TRNG operation.
